# IRS-1 increases TAZ expression and promotes osteogenic differentiation in rat bone marrow mesenchymal stem cells

**DOI:** 10.1242/bio.036194

**Published:** 2018-12-15

**Authors:** Na Wang, Peng Xue, Ziyi Li, Yukun Li

**Affiliations:** 1 Department of Endocrinology, The Third Hospital of Hebei Medical University, 139 Ziqiang Road, Shijiazhuang 050051, Hebei Province, China; 2 Key Orthopaedic Biomechanics Laboratory of Hebei Province, 139 Ziqiang Road, Shijiazhuang 050051, Hebei Province, China

**Keywords:** IRS-1, TAZ, PI3K-Akt, BMSCs, Osteogenic differentiation

## Abstract

Whether insulin receptor substrate 1 (IRS-1) inhibits or promotes the osteogenic proliferation and differentiation *in vitro* remains controversial. Transcriptional co-activator with PDZ-binding motif (TAZ) plays a vital role in the osteogenesis of bone marrow mesenchymal stem cells (BMSCs), and strongly activates the expression of the osteogenic differentiation markers. In this study, we found that IRS-1 and TAZ followed similar increasing expression patterns at the early stage of osteogenic differentiation. Knocking down IRS-1 decreased the TAZ, RUNX2 and OCN expression, and overexpressing IRS induced the upregulation of the TAZ, RUNX2 and OCN expression. Furthermore, our results showed that it was LY294002 (the PI3K-Akt inhibitor), other than UO126 (the MEK-ERK inhibitor), that inhibited the IRS-1 induced upregulation of TAZ expression. Additionally, SiTAZ blocked the cell proliferation in G1 during the osteogenic differentiation of BMSCs. Taken together, we provided evidence to demonstrate that IRS-1 gene modification facilitates the osteogenic differentiation of rat BMSCs by increasing TAZ expression through the PI3K-Akt signaling pathway.

This article has an associated First Person interview with the first author of the paper.

## Abstract (precis)

**Summary:** Stem cell therapy has become a promising approach for bone tissue engineering. Our study reveals that IRS-1 gene modification of stem cells upregulates TAZ expression and osteogenesis.

## INTRODUCTION

Bone tissue could be continuously regenerated, but it often fails when the healing capacity is compromised (Lupsa and Insogna, 2015). Many clinical conditions, such as osteoporosis or diabetes, make bone remodeling difficult. Recently, stem cell replacement therapy has become a promising approach for bone tissue engineering (Ye et al., 2017). Bone marrow mesenchymal stem cells (BMSCs) are one of the ideal sources of the seed cell for bone tissue repair due to their multi-potent potential to differentiate into numerous cell types, but there are still many questions about the control of the mesenchymal stem cell fate during its differential process (van Zoelen et al., 2016). The commitment of BMSCs toward a specific cell type, either of osteogenic or adipogenic lineage, mostly depends on the change of the cell micro-environment, specifically concerning transcriptional regulators (Ullah et al., 2015). Transcriptional co-activator with PDZ-binding motif (TAZ), a transcriptional modulator, is one of such regulators that have key roles in cell proliferation, differentiation and stem cell self-renewal (Konishi et al., 2018; Tang et al., 2016; Xiao et al., 2018). Although TAZ does not harbor a DNA-binding domain, it could interact with different kinds of transcription factors to activate or repress specific gene expression, which might influence cell functions (Kim et al., 2016; Panciera et al., 2017; Xue et al., 2013; Zhou et al., 2016). It has also been reported that TAZ could be combined with peroxisome proliferator-activated receptor γ (PPARγ) to inhibit adipogenesis or RUNX2 to facilitate osteogenesis (Kim et al., 2016; Xue et al., 2013; Zhou et al., 2016). Besides, we have discovered that exogenous stimuli, such as insulin/IGF-1, could increase TAZ expression to promote osteogenic differentiation in BMSCs (Xue et al., 2013). However, it is of interest whether or not TAZ plays an important role in insulin/IGF-1 pathways during the BMSCs' osteoblastogenesis.

Insulin receptor substrate (IRS)-1 and -2 are the major signaling adapters in insulin/IGF-1 pathways and are rapidly phosphorylated on multiple tyrosine residues after ligand stimulation. Phosphorylated IRS-1 binds to proteins containing Src homology (SH)-2 domains, then stimulates a variety of downstream pathways that regulate cell proliferation and differentiation (Guo et al., 2016; Ma et al., 2015). It was reported that IRS-1-knockout mice exhibited low bone mineral density or severe osteopenia, with low bone turnover (Ogata et al., 2000). Also, Guo et al. (2016) have uncovered a temporal regulation of BMSCs' differentiation/bone formation, controlled via IRS-1/miR-342 mediated regulation of Col1a2 expression in IRS-1 deficient mice. Our previous studies also showed that IGF-1, IRS-1 and IRS-2 were reduced in ovariectomy-induced osteoporotic rats in liver, skeletal muscle, kidney and bone; moreover, the decreases were more rapid and profound in bone compared to other tissues (Li et al., 2013). At the cellular level, our team proved that IRS-1 promotes cell proliferation, bone formation and mineralization in pre-osteoblasts (Ma et al., 2015). Additionally, IRS-1 was reported to play an important role in facilitating osteogenic differentiation (Xi et al., 2016). However, the underlying mechanisms deserve further studies.

In addition to the transcriptional networks which control the lineage-specific differentiation program, we have revealed a new interaction between IRS-1 and TAZ, which may be involved in osteogenic differentiation and skeletal formation. Note that IRS-1 increased TAZ expression and facilitated osteoblatogenesis, and that regulation was mostly mediated by the PI3K-Akt pathway rather than the MEK-ERK pathway. This study also pointed to a close link between the insulin signaling pathway and the TAZ/RUNX2 transcriptional complex during osteoblast maturation.

## RESULTS

### Gene expression of IRS-1 was associated with TAZ during osteogenic differentiation of passage 3 BMSCs

**Fig. 1.**
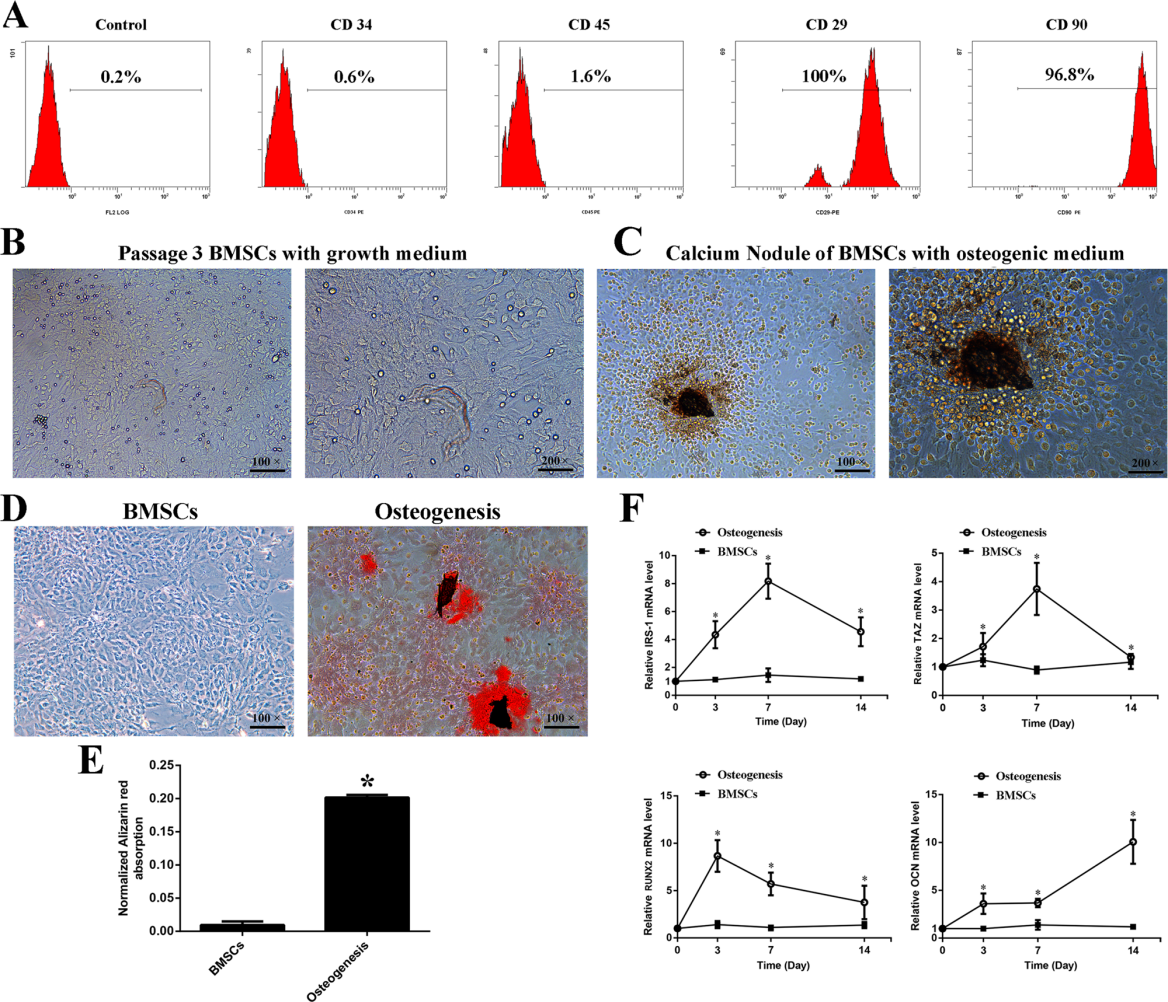
**The verification of the BMSCs and the change of IRS-1, TAZ, RUNX2 and OCN mRNA expression during osteogenic differentiation.** (A) The BMSCs were isolated from bone marrow, cultured in vitrol and verified by flow cytometry for hematopoietic surface markers CD34 (0.6%) and CD45 (1.6%), and BMSC surface markers CD29 (100%) and CD90 (96%). (B,C) Passage 3 BMSCs were cultured with growth medium or (C) osteogenic medium for 14 days. (D) Alizarin Red staining was performed and (E) the quantification by cetylpyridinium chloride dissolution was presented after inducing osteogenic medium for 14 days. (F) The mRNA expression of IRS-1, TAZ, RUNX2 and CON in BMSCs cultured in osteogenic medium for 3, 7 and 14 days were measured by fluorogenic quantitative PCR. (s.d.±mean; *n*=3) *P<0.05 versus control group.

Passage 3 BMSCs were positive for cell lineage markers such as CD29 (100%) and CD90 (96.8%), and negative for CD34 (0.6%) and CD45 (1.6%) (Fig. 1A). Additionally, we verified the BMSCs with Alizarin Red staining (AR-S) after 14 days of osteogenic differentiation (Fig. 1B-E). The real-time RT-PCR results showed that IRS-1 expression was increased in a time-dependent manner during the process of the BMSCs' osteogenic differentiation (Fig. 1F). The IRS-1 expression increased after treatment with osteogenic differentiation medium from 3 to 7 days, and decreased from 7 to 14 days. Further, we have discovered a similar trend for TAZ expression during osteogenic differentiation (Fig. 1G). While RUNX2 expression increased at the beginning of osteogenic differentiation and then decreased; mRNA levels were always significantly higher than those of the control (Fig. 1H). Consistent with a previous report (Born et al., 2012), a delayed increase of OCN expression was also confirmed during osteogenic differentiation (Fig. 1I). These data indicated that IRS-1 was upregulated during the process of osteogenic differentiation and associated with osteogenesis in BMSCs, and that the underlying mechanism might have involved the upregulation of TAZ expression, which then increased the expression of RUNX2 and OCN.

### SiIRS-1 transfection inhibited osteogenic differentiation as well as decreasing TAZ expression

**Fig. 2.**
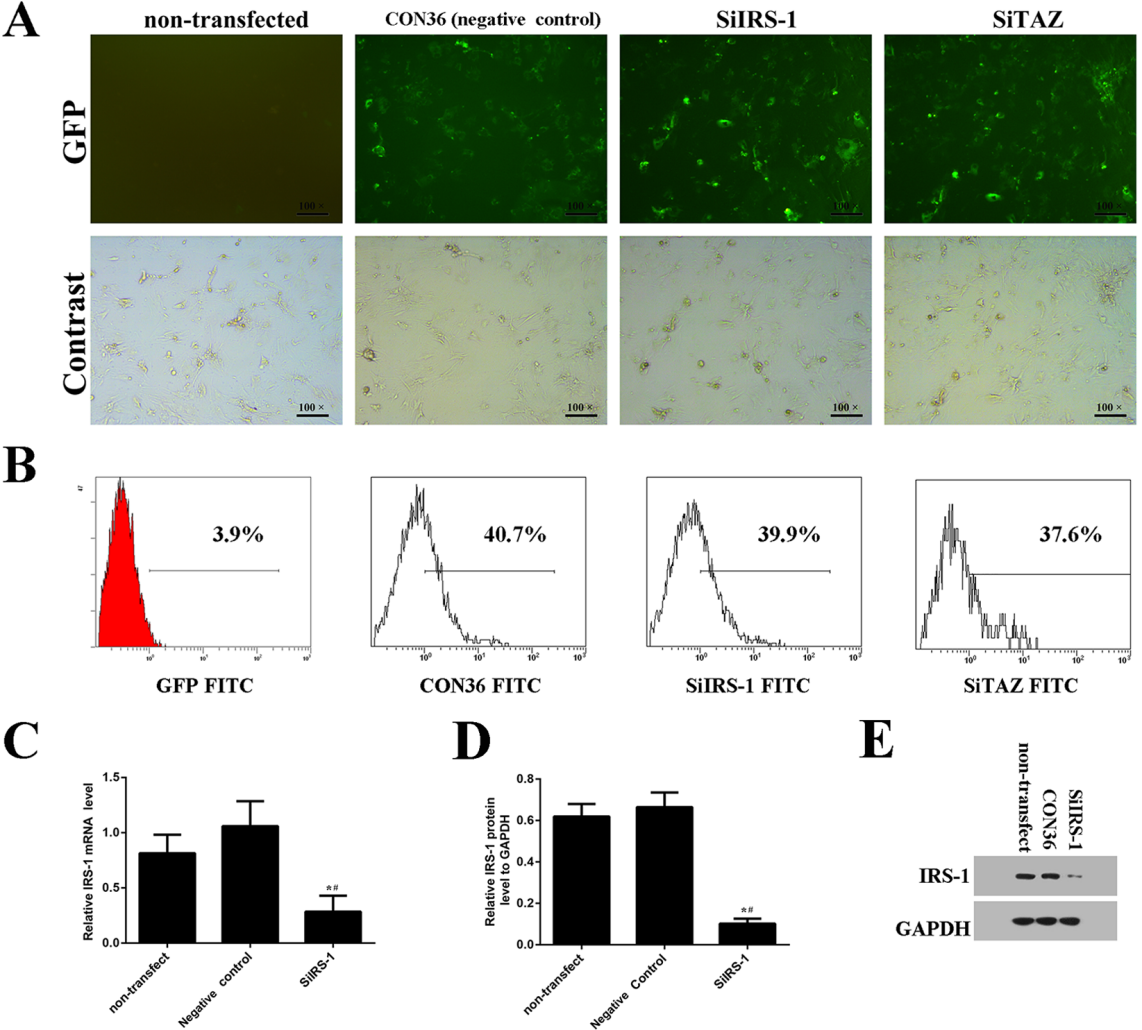
**The transfection efficiency of SiIRS-1, SiTAZ and the vacant plasmid as negative control during osteogenic differentiation of BMSCs.** (A) Seventy-two hours after transfection, several GFP+ cells were observed and counted under fluorescence microscopy. (B) Transfection efficiency was detected by the flow cytometer. (C-E) IRS-1 expression was analyzed using fluorogenic quantitative PCR (C,D) and western blotting (E). (s.d.±mean; *n*=3) *P<0.05 versus non-transfected group; ^#^*P*<0.05 versus negative control group.

**Fig. 3.**
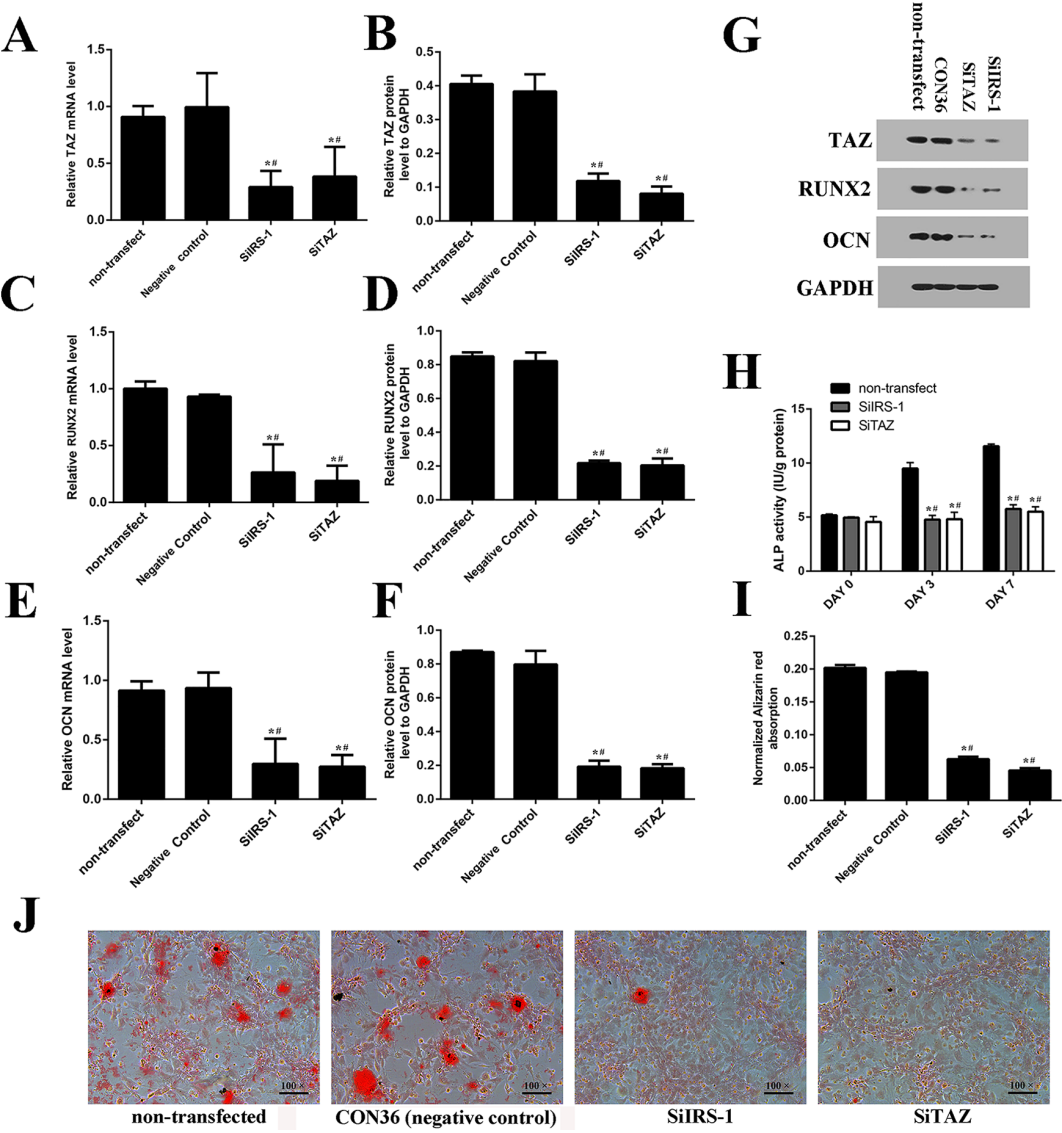
**SiIRS-1 transfection inhibited osteogenic differentiation as well as decreasing TAZ expression.** (A-G) SiIRS-1 transfection inhibited the expression of TAZ, RUNX2 and OCN; measured by fluorogenic quantitative PCR and western blot. (H) The ALP activities were measured at 0, 3 and 7 days after osteogenic medium introduction. (I,J) Alizarin Red staining was performed and the normalized Alizarin Red absorption was presented after inducing osteogenic medium for 14 days. (s.d.±mean; *n*=3) *P<0.05 versus non-transfected group; ^#^*P*<0.05 versus negative control group.

We knocked down IRS-1 expression using plasmids containing the SiIRS-1 sequence. Green fluorescent protein positive (GFP+) cells were also observed with fluorescence microscopy (Fig. 2A). Transfection efficiency of the plasmid and its negative control were high enough to be comparable (Fig. 2A,B). Both real-time RT-PCR and western blot analyses suggested that SiIRS-1 or SiTAZ significantly decreased their expression compared with the negative control and non-transfected cells (Figs 2C-E and 3A,B,G). Furthermore, transfection with the SiIRS-1 plasmid blocked the upregulation of TAZ, RUNX2 and OCN expression (Fig. 3A-G) after the introduction of osteogenic medium, which was confirmed not only in the ALP activity assay, but also in the AR-S (Fig. 3H-J). Firstly, the ALP activities were significantly reduced in SiIRS-1 group compared with the vacant plasmid (CON36) treatment group, and there was no significant difference of the ALP activities among the SiIRS-1 and SiTAZ treatment groups (Fig. 3H). Secondly, the AR-S results were consistent with the ALP activity assay results (Fig. 3I,J).

### IRS-1 overexpression promoted osteogenic differentiation as well as increasing TAZ expression

**Fig. 4.**
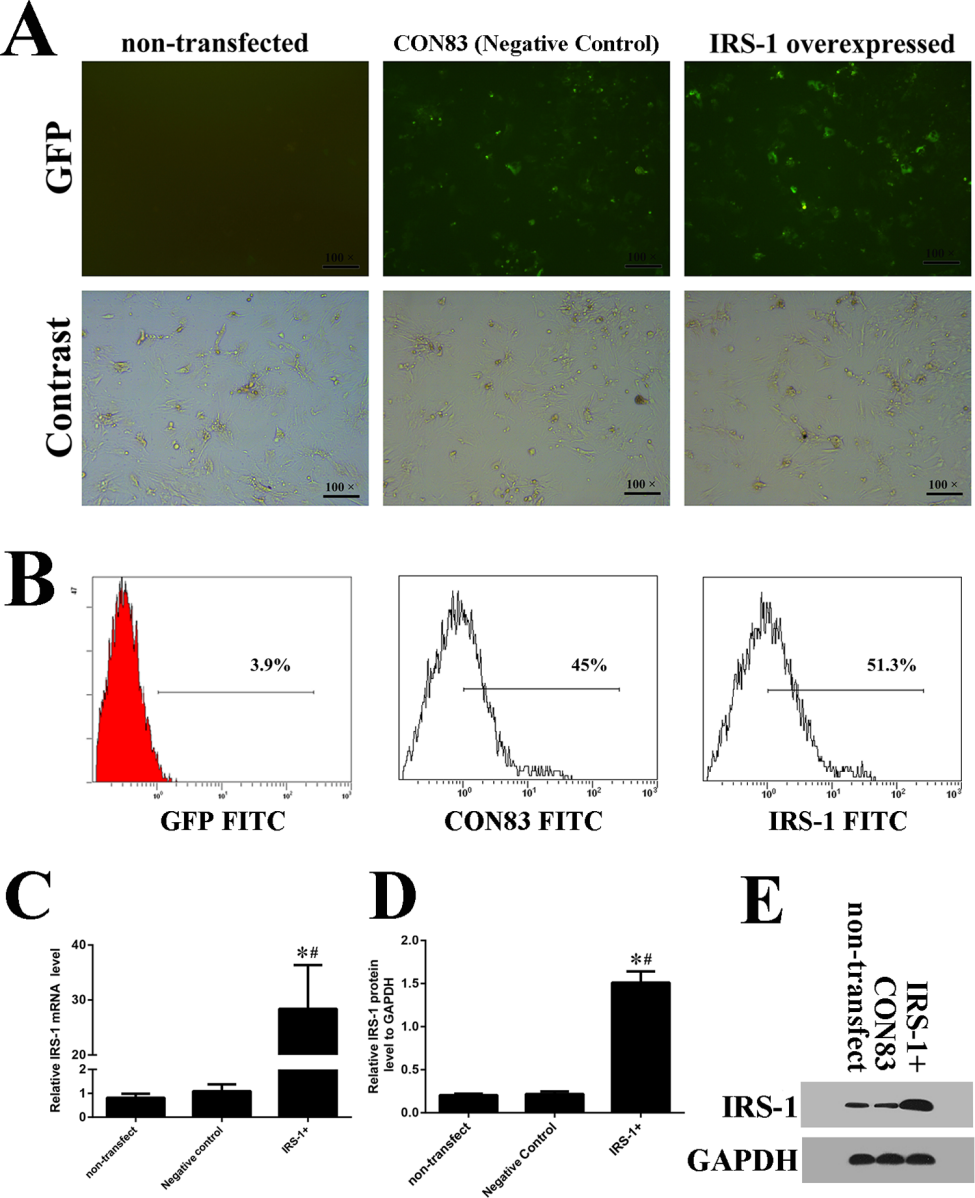
**The transfection efficiency of overexpressed IRS-1 and the vacant plasmid as negative control on the osteogenic differentiation of BMSCs.** (A) Seventy-two hours after transfection, several GFP+ cells were observed and counted under fluorescence microscopy. (B) Transfection efficiency was detected by the flow cytometer. (C-E) IRS-1 expression was analyzed using fluorogenic quantitative PCR and (C,D) western blotting (E). (s.d.±mean; *n*=3) *P<0.05 versus non-transfected group; ^#^*P*<0.05 versus negative control group.

**Fig. 5.**
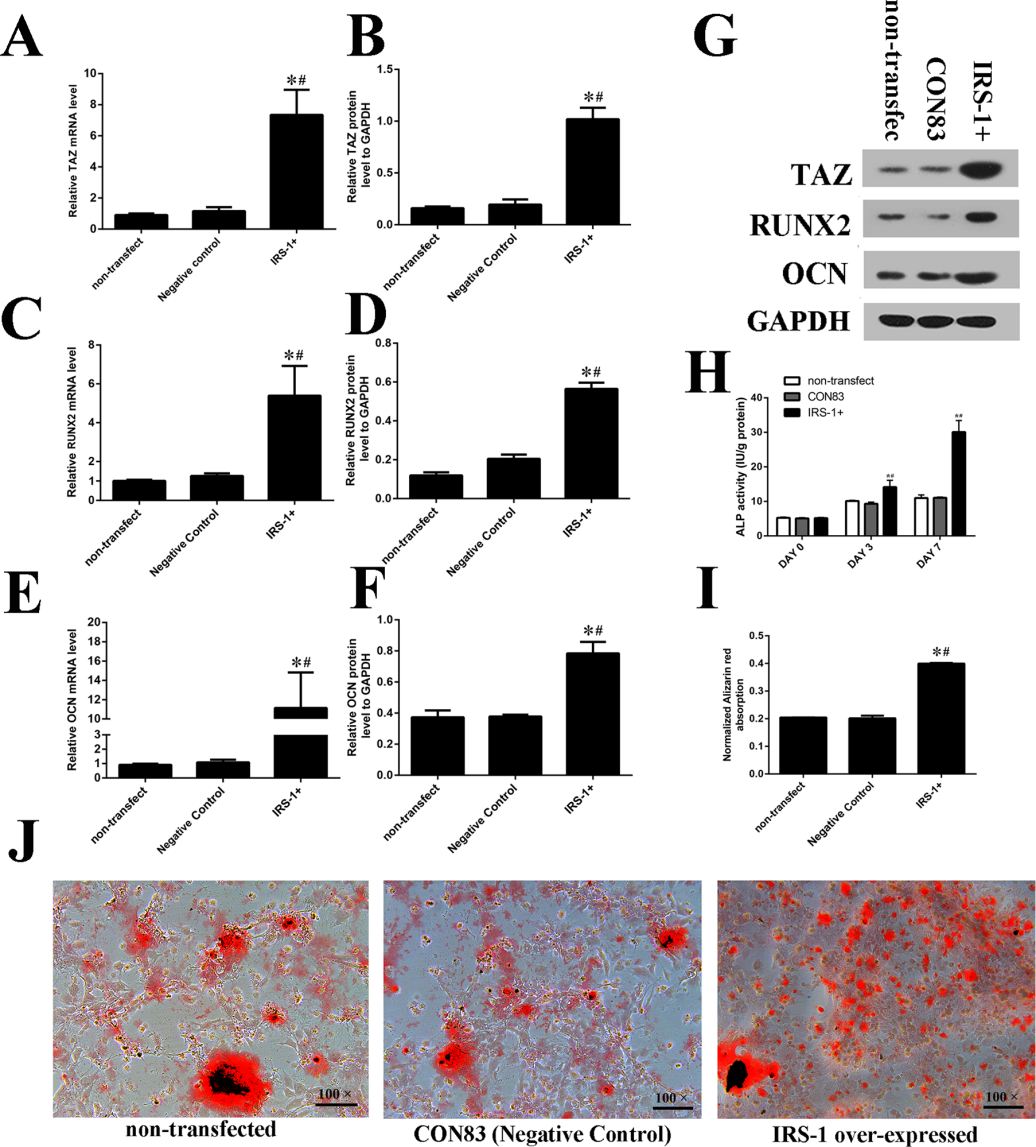
**IRS-1 overexpression promoted osteogenic differentiation as well as increasing TAZ expression.** (A-G) IRS-1 overexpression increased the TAZ, RUNX2 and OCN expression which were measured by fluorogenic quantitative PCR and western blotting. (H) The ALP activities were promoted in IRS-1+ group at 0, 3 and 7 days after osteogenic medium introduction. (I,J) Alizarin Red staining and the normalized Alizarin Red absorption were consistent with the ALP results which were presented after inducing osteogenic medium for 14 days. (s.d.±mean; *n*=3) *P<0.05 versus non-transfected group; ^#^*P*<0.05 versus negative control group.

To verify the effects of IRS-1 on TAZ expression, BMSCs were transfected with the expression vector for IRS-1 (Fig. 4A). Seventy-two hours after transfection, several GFP+ cells were observed and counted under fluorescence microscopy (Fig. 4A,B). Transfection efficiency was detected by the flow cytometer and a similar high efficiency was discovered. Both real-time RT-PCR and western blot analysis results showed a higher IRS-1 expression in the IRS-1 overexpression group than in the negative control group and the non-transfected group (Fig. 4C-E). Specifically, the expression of TAZ, RUNX2 and OCN (Fig. 5A-G) were markedly increased by IRS-1 overexpression compared with their expression in cells transfected with vacant plasmid and non-transfected cells as well. High expression of IRS-1 significantly improved ALP activity compared with the negative control group and non-transfection group (Fig. 5H) 7 days post-transfection. Moreover, AR-S demonstrated an increase in the volume of mineralized nodules when IRS-1 was overexpressed (Fig. 5I,J). These results indicated that IRS-1 increased TAZ expression and facilitated osteogenic differentiation of BMSCs.

### IRS-1 increased TAZ expression mediated by the PI3K-Akt pathway rather than the MEK-ERK pathway

**Fig. 6.**
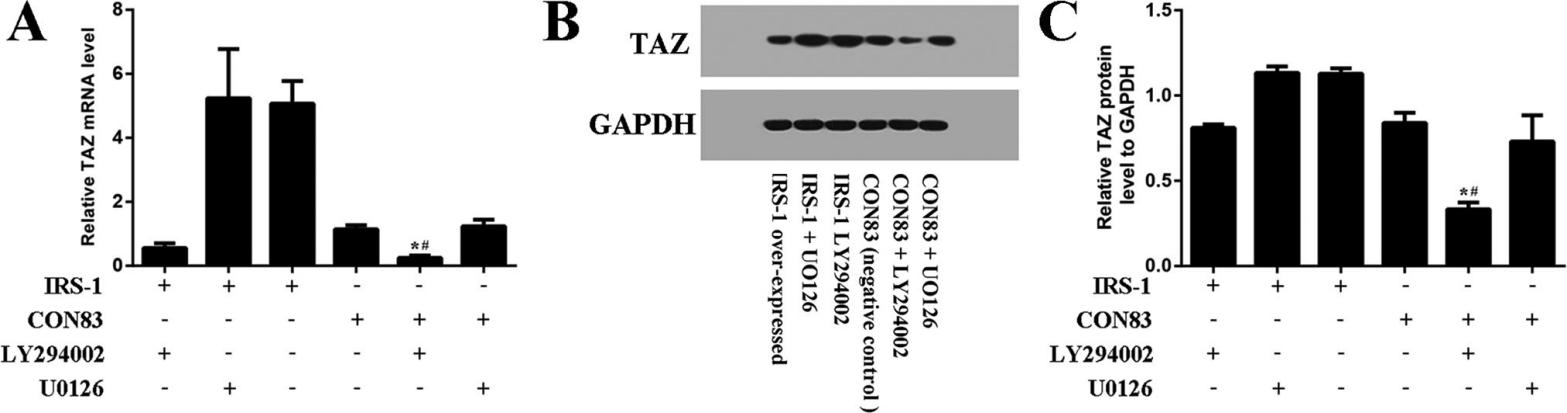
**IRS-1 increased TAZ expression mediated by the PI3K-Akt signaling pathway.** (A) Real-time RT-PCR and (B,C) western blot analyses for the relative TAZ mRNA levels at day 3 after the introduction of osteogenic medium. (C) Bar graphs show the means±s.d. of the relative protein levels from three independent experiments (s.d.±mean; *n*=3) *P<0.05 versus LY294002+IRS-1 group; ^#^*P*<0.05 versus CON83, the negative control group.

In BMSCs, UO126 and LY294002 are the inhibitors for the MEK–ERK pathway and the PI3K-Akt pathway, respectively. The real-time RT-PCR results (Fig. 6A) revealed that TAZ mRNA levels were significantly higher in the pCMV-IRS-1+LY294002 treatment group than those in the CON83+LY294002 treatment group. TAZ mRNA levels were then significantly reduced in the pCMV-IRS-1+LY294002 treatment group compared with the pCMV-IRS-1 treatment group. Also, TAZ mRNA levels in the CON83+LY294002 treatment group were significantly lower than that in the CON83 transfected group. However, there was no significant difference in TAZ mRNA levels between the pCMV-IRS-1+UO126 and pCMV-IRS-1 treatment groups, or between the CON83+UO126 and CON83 treatment groups. Furthermore, western blot analysis results were in line with real-time RT-PCR results (Fig. 6B). These results indicated that PI3K-Akt pathway was involved in the regulation of IRS-1 on TAZ during osteogenesis.

### IRS-1 has no effect on the proliferation of BMSCs during osteogenic differentiation

**Fig. 7.**
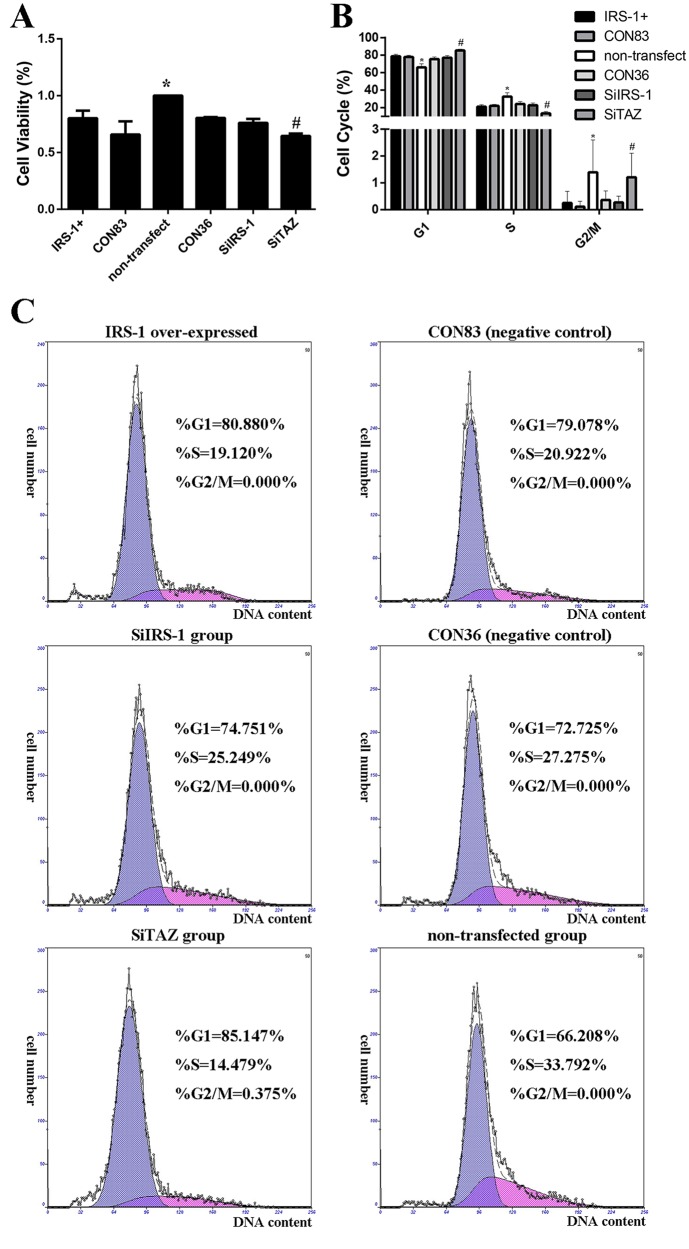
**Effects of IRS-1 and TAZ** **on the mesenchymal stem cell proliferation during its osteogenesis.** (A) Cell viability was measured 4 days post-transfection with osteogenic medium for 3 days. (B) Bar graphs show means±s.d. from three independent experiments. (C) Representative flow cytometry experiment results are presented in each group. The percentages of cells in the G1, S and G2 phases of the cell cycle are shown in each individual graph. (s.d.±mean; *n*=3) *P<0.05 versus any other groups. ^#^*P*<0.05 versus SiIRS-1 or CON36 or non-transfected group.

Preliminarily, the MTT assays showed that the cell viability of all transfected cells was lower than that of non-transfected cells (Fig. 7A). Of interest, cell viability in the SiTAZ group was significantly decreased compared with its negative control group (Fig. 7A). However, there were no significant differences among the other transfected groups, whether in the IRS-1 overexpression group or the SiIRS-1 group compared with their respective negative control groups (Fig. 7A). In parallel with this analysis, cell cycles measured by the flow cytometer also indicated that IRS-1 has no effect on the proliferation of BMSCs during osteogenic differentiation, but SiTAZ might inhibit the self-renewal of BMSCs by blocking the cell cycle in G1 (Fig. 7B,C). The percentage of cells in the G1 phase was significantly increased in SiTAZ-transfected cells compared with non-transfected cells and transfected cells with vacant plasmids as negative control, suggesting an inhibiting effect of SiTAZ on cell cycles moving to G2/M. The percentage of cells in the G2/M phase of the whole cell cycle was very low: no more than 3%. In Fig. 7B, we can see that G2/M population was changed by TAZ silencing compared with non-transfected samples, but not by much.

**Fig. 8.**
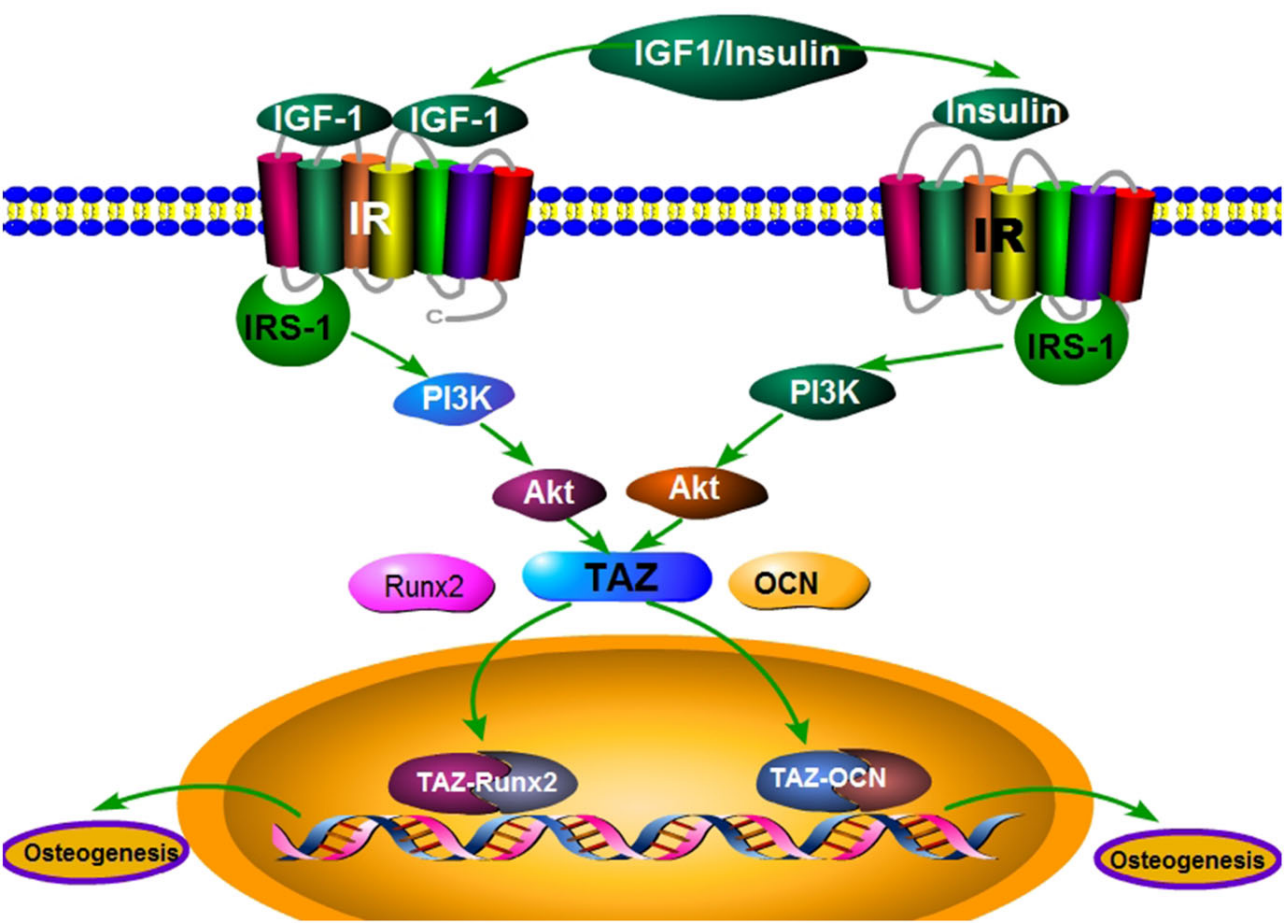
**An illustration of the signaling pathways involved in the role of IRS-1 in promoting BMSCs' osteogenic differentiation.**

In summary, increased TAZ expression by IRS-1 might be partially involved in the regulation of the PI3K-Akt pathway, specifically to induce the upregulation of the osteogenic markers RUNX2 and OCN, and facilitate osteoblatogenesis of BMSCs (Fig. 8).

## DISCUSSION

Osteoporosis is a bone metabolic disease which is characterized by a systemic impairment of bone formation and an increase in bone resorption, leading to a high risk of fragility fractures (Lupsa and Insogna, 2015). Gene modification of stem cells to facilitate osteogenic differentiation is an effective approach to treat osteoporosis, and *in vitro* culture of rodent BMSCs is a key research tool in bone biology. In this study, we show a possible role for IRS-1 in the mesenchymal stem cell fate determination to osteoblasts. In BMSCs, IRS-1 could significantly promote osteogenic differentiation by increasing TAZ expression, which makes IRS-1 a potential therapeutic target for mitigating osteoporosis by stem cell therapy in the near future.

As we know, IRS-1 acts as a docking protein, coordinating with hormones binding to the receptor and downstream signaling effectors containing SH2 domains to regulate cell metabolism, growth and differentiation (Xi et al., 2017; Zhao et al., 2017). It has been reported that the number of osteoblasts, and therefore the rate of bone formation, was reduced in mice that specifically lacked insulin receptor (IR) expression in osteoblasts (Fulzele et al., 2010). However, the effect of IRS-1 on bone metabolism remains a controversial issue. In contrast to the well-established model that transcriptional networks control the lineage-specific maturation program in multicellular organisms, we have uncovered a protein amplification between IRS-1 and TAZ that is required for osteogenesis of BMSCs. We firstly found a similar trend of the IRS-1 mRNA levels with the TAZ mRNA levels during osteogenic differentiation. No matter the IRS-1 down- or up-expression, TAZ mRNA and protein levels could change accordingly, as well as the osteogenic differentiation markers.

Previously, we discovered the pivotal role of TAZ in the osteogenic differentiation of BMSCs derived from rat bone marrow (Xue et al., 2013). It has a single WW domain, and the WW domain of TAZ binds strongly to the sequence motif Pro-Pro-X-Tyr (Panciera et al., 2017). This motif can be discovered within the regulatory region of RUNX2, and TAZ could strongly activate RUNX2-driven genes during the terminal osteogenic differentiation (Kim et al., 2016; Zhou et al., 2016). As has been previously reported, deletion of TAZ in zebrafish results in a lack of ossification and ventral curvature (Hong et al., 2005). In this study, we confirmed that SiTAZ significantly downregulates the expression of RUNX2 and OCN in the osteogenic differentiation process. Similar effects could be seen after cells were transfected with SiIRS-1 plasmid, indicating a significant association of IRS-1 with TAZ to influence bone formation *in vitro*. Actually, SiIRS-1 could downregulate the expression of TAZ during osteogenic differentiation. To further investigate the effects of IRS-1 on TAZ, we transfected cells with pCMV-IRS-1, and found upregulation of TAZ induced by IRS-1 overexpression both at mRNA levels and protein levels, confirming the link between IRS-1 and TAZ expression.

Except for TAZ expression, the upregulation of RUNX2 and OCN expression in the IRS-1 overexpression group also suggested that IRS-1 could enhance the RUNX2- and OCN-driven osteogenic gene expression accordingly. At first, we discovered that IRS-1 and RUNX2 mRNA levels were increased at the early stage of osteogenesis, while the OCN mRNA levels presented a delayed increase, as shown in previous studies (Born et al., 2012; Zhang et al., 2016). In addition, the mRNA and protein levels of RUNX2 and OCN were both significantly increased after 3 days' induction of the osteogenic medium in the IRS-1 overexpression group. Therefore, we inferred that IRS-1 could increase RUNX2 and OCN expression at the early stage of osteogenic differentiation. In fact, RUNX2 and OCN are two key osteoblast markers in the process of osteogenic differentiation. RUNX2 is known to be a critical and early regulator of osteogenic development. RUNX2 knockout in mice resulted in complete depletion of bone formation (Kidwai et al., 2016). OCN, known as bone-carboxyglutamic acid-containing protein (BGLAP) preferentially expressed by osteoblasts, is the most abundant non-collagenous bone matrix protein. Therefore, OCN was often used as a late marker for bone formation (Tsao et al., 2017). Consistently, in this study, we proved that IRS-1 positively correlated with both RUNX2 and OCN expression after 3 days’ induction of the osteogenic medium.

As we know, PI3K-Akt is indispensable for cell proliferation and differentiation and it is the central mediators for insulin/IGF1 signaling (Luo et al., 2017; Song et al., 2017; Wang et al., 2016a). Our team has discovered that IRS-1 activated the PI3K-Akt signaling pathway to enhance the proliferation of the primary rat osteoblasts (Ma et al., 2015). Although we found that the increased TAZ expression induced by IGF1 is mostly mediated by the MEK-ERK pathway rather than the PI3K-Akt signaling pathway in our previous research (Xue et al., 2013), Xie et al. (2015) reported that inhibition of the PI3K-Akt pathway could attenuate the activation of TAZ expression in lung cancer cells. Our results revealed that the PI3K-Akt pathway inhibitor, LY294002, offsets the IRS-1 induced upregulation of TAZ expression, which points to a link between the IRS-1/PI3K-Akt signaling pathway and the TAZ/RUNX2 transcriptional complex during osteoblast maturation.

As for the effects of IRS-1 on cell proliferation, it is well recognized that IRS-1 promotes proliferation in many cell types (Lai et al., 2016; Machado-Neto et al., 2011). Lai et al. (2016) found that the promotion of hepatocellular carcinoma (HCC) cell proliferation caused by miR-384-in was counteracted by silencing IRS1 expression with siRNAs, which provided convincing evidence that miR-384 exerted a suppressive effect on HCC cell proliferation through the direct inhibition of IRS1 expression. Even in the human leukemic cell line K562, it was demonstrated that knockdown of IRS-1 reduced proliferation though downregulation of the Akt/mTOR and MAPK pathways (Machado-Neto et al., 2011). As well, our team discovered that IRS-1 enhanced the proliferation of the primary rat osteoblasts (Xue et al., 2013). However, it is unclear whether IRS-1 facilitates osteogenesis through its positive effect on the proliferation of the osteoblasts or not. Our results discovered that IRS-1 over- or under-expression has no effect on the cell cycles during osteogenesis; but transfection might reduce the cell viability by using the liposome in transfection procedure.

Currently, it is considered that the Hippo transducer TAZ could not only promote cell growth, but also inhibit Celastrol-induced cell apoptosis to impact cell expansion (Wang et al., 2016b; Yang et al., 2016; Ye et al., 2017). Wang et al. (2016b) reported that overexpression of TAZ could promote cell growth and partially restore Celastrol-induced cell apoptosis *in vitro* and *in vivo*. Consistent with previous research, we knocked down TAZ expression with SiTAZ plasmid and found that cell viability was significantly lower than in the vacant plasmid group and that SiTAZ inhibited the cell cycles in G1, indicating that TAZ does affect BMSCs' proliferation, even in the process of osteogenic differentiation. This might be a new approach for TAZ to facilitate skeletal formation. In the near future, we will further study the underlying mechanism of TAZ induced BMSC proliferation during osteogenic differentiation.

## CONCLUSIONS

Stem cells are dependent on their ability to maintain their pool whilst also being able to respond to physiological and pathological conditions to repair and renew tissues throughout the lifetime of the organism. In summary, our data demonstrates a close interaction between IRS-1 and TAZ during bone formation, indicating that IRS-1 may be a promising target for the development of new therapeutic treatments for osteoporosis.

## MATERIALS AND METHODS

### Cell culture and differentiation

All animal experiments in this study were approved by the Local Committee of Animal Use and Protection of the Third Hospital of Hebei Medical University (China). A total of 20 four-week-old male Sprague-Dawley rats were obtained from the Centre of Laboratory Animal Science at Hebei Medical University. BMSCs were isolated from the rats and plated at a concentration of 5×10^6^/cm^2^ in Dulbecco's modified Eagle's medium (DMEM-LG; Gibco, USA) containing 12% fetal bovine serum (FBS; Gibco) and 1% penicillin-streptomycin (Gibco). Medium was changed every 3 days so that non-adherent cells could be removed and the BMSCs were purified after passage 3 BMSCs. Cells were allowed to expand in a 37°C incubator with 5% CO_2_ until confluence reached 75-80%. In order to identify the expression levels of classical cells' surface markers, passage 3 BMSCs were stained with anti-CD34, anti-CD45, anti-CD29 and anti-CD90 (all from Thermo Fisher Scientific) in the presence of phycoerythrin (PE) and sorted by fluorescence-activated cell sorting. Representative histograms of CD29 and CD90 positive and CD34 and CD45 negative expression in the passage 3 BMSCs were employed in the subsequent experiments. Osteoblastic differentiation was induced by culturing cells in the osteogenic medium [DMEM supplemented with 12% FBS, 10 nM dexamethasone (Sigma-Aldrich), 10 mM β-glycerophosphoric acid (Sigma-Aldrich), and 50 μg/ml ascorbic acid (Sigma-Aldrich)].

### Plasmids transfection

We successfully constructed an expression vector for IRS-1 (pCMV-IRS-1) in a previous study (Ma et al., 2015). The plasmids with small interfering RNAs (SiRNAs) against rat IRS-1 (SiIRS-1) and TAZ (SiTAZ) expression were designed and synthesized by Shanghai Genechem Corporation (Shanghai, China). Relatively vacant plasmids were used as negative controls for pCMV-IRS-1 with a name of CON83, as well as for the SiIRS-1 or SiTAZ group (same negative control with a name of CON36). The difference between CON36 and CON83 was their antibiotic resistance. Cultured cells were transfected with a plasmid using Lipofectamine 3000 (Thermo Fisher Scientific) after reaching ∼80% confluence, in accordance with manufacturer's instructions. After being transfected for 24 h, the cells were then switched to growth medium or osteogenic medium for osteogenic differentiation. The transfection efficiency of each plasmid was measured by flow cytometry (Epics XL, Beckman Coulter Corporation, USA) 4 days post-transfection. Meanwhile, transfected cells expressing GFP reporter were analyzed under a fluorescence microscope (Leica, Wetzlar, Germany). Furthermore, the expression of IRS-1 and TAZ in different groups was assessed using real-time RT-PCR and western blot analysis.

### Cell viability assay

72 h post-transfected cells were seeded at a density of 5×10^4^ cells/100 μl/well in 96-well plates and incubated for cell viability assay. After 24-48 h cell attachment, 20 μl of freshly prepared MTT (5 mg/ml; Solarbio) was added and the plates were incubated at 37°C for another 4 h to form crystals. Then, 150 μl dimethyl sulfoxide (DMSO) was added for 10 min to fully dissolve the crystals. Finally, the absorbance of each well was measured at a wavelength of 490 nm using a microplate spectrophotometer (BioTek Instruments, San Jose, USA).

### Cell cycle analysis

To identify the effect of IRS-1 on cell cycles, 3 day post-transfected cells were harvested and fixed with 70% ethanol. After washing in PBS, cells were stained with Propidium Iodide (Sigma-Aldrich) (5 mg/ml) for 30 min in the dark at 4°C. Fluorescence was measured with the flow cytometer equipped with a 570 nm argon ion laser (Epics XL, Beckman Coulter Corporation, USA) and the data were analyzed using the MultiCycle AV software.

### ALP activity assay

Cells were seeded in 24-well plates (Costar) for ALP activity assay. Cells were harvested and re-suspended in 250 μl culture supernatants, followed by cell breaking with an ultrasound breaker. After centrifugation, the ALP activities in the cell supernatants were quantified using an ALP Detection Kit (Nanjing Jiancheng Biotech Institute, China) according to the manufacturer's instructions and a spectrophotometer at a wavelength of 520 nm using the microplate spectrophotometer. Each value was normalized with the protein concentration.

### Alizarin Red staining

Cells were seeded in 35-mm plastic dishes (Costar) for AR-S. Cells were washed twice with PBS and fixed with 4% paraformaldehyde (Solarbio, China) at room temperature for 20 min. Then the dishes were washed three times with distilled water and incubated with 0.1% Alizarin Red (Sigma-Aldrich) at 37°C for 30 min. Cells were then washed thoroughly with distilled water and the images were acquired using the microscope (Leica). Then, AR was de-stained with 10% cetylpyridinium chloride (Sigma-Aldrich) in PBS for 30 min at room temperature. The calcium concentrations were determined according to the absorbance at 562 nm using a standard calcium curve prepared in the same solution.

### Real-time reverse transcription-polymerase chain reaction (real-time RT-PCR)

Total RNA was extracted using TRIzol^®^ reagent (Ambition). Total RNA (2 μg) was reversed-transcribed into cDNA using a GoScript™ Reverse Transcription System kit (Promega, USA). Real-time RT-PCR was performed using GoTaq^®^ qPCR Master Mix (Promega) in a quantitative PCR System (Corbett) with a total volume of 20 μl containing 2 μl of the cDNA products, 10 μl of GoTaq qPCR Master Mix (2×) (Promega), 0.6 μl of forward primer, 0.6 μl of reverse primer and 6.8 μl of Nuclease-Free Water. All primers were synthesized by the same manufacturer (Invitrogen). Real-time RT-PCR reaction conditions were: 95°C for 10 min, followed by 40 cycles of 95°C for 15 s and 60°C for 1 min. The relative expression was calculated according to the ratio of the copy numbers of the target genes (IRS-1, TAZ, RUNX2 and OCN) to the housekeeping gene GAPDH in each sample. The relative gene expression values were evaluated by the 2^−△△Ct^ method. Sense and antisense primers were listed as follows:

GAPDH: 5′-AGTTCAACGGCACAGTCAAGG-3′, 5′-AGCACCAGCATCACCCCAT-3′;

IRS-1: 5′-CCTGACATTGGAGGTGGGTC-3′, 5′-TTACCACCACCGCTCTCAAC-3′;

TAZ: 5′-ATGTTGACCTCGGGACTTTGG-3′, 5′-GAGGAAGGGCTCGCTTTTGT-3′;

RUNX2: 5′-GCACCGACAGCCCCAACTT-3′, 5′-CCACGGGCAGGGTCTTGTT-3′;

OCN: 5′-CAGGAGGGCAGTAAGGTGG-3′, 5′-CAGGGGATCTGGGTAGGG-3′.

### Western blot analysis

Cells were seeded in 60 mm plastic dishes (Costar) for total protein isolation. Proteins were separated by 12% SDS-PAGE and transferred to a PVDF membrane using a semidry transfer apparatus (Hoefer) for 1.5 h at room temperature. Membranes were blocked with 5% milk in TBST for 2 h at 37°C, and incubated with primary antibodies against IRS-1 (1:1000, Cell Signaling, USA), TAZ (1:1000, Cell Signaling), RUNX2 (1:200, Boster, China), OCN (1:200, Boster) or GAPDH (1:3000, Bioworld, USA) at 4°C overnight. Membranes were incubated with IRDye800^®^ conjugated secondary antibody (1:20,000, Rockland, USA) for 1 h at 37°C, following scanning with the Odyssey Infrared Imaging System (Li-COR Biosciences). Then the integrated intensity for each detected band was determined with Image J, v.1.46.

### Inhibitor study

10 μM MEK-ERK inhibitor U0126 (Beyotime Institute of Biotechnology, China) and PI3K-Akt inhibitor LY294002 (Beyotime Institute of Biotechnology) were added simultaneously into osteogenic medium for 24 h after transfection. Then the expression of IRS-1 and TAZ were detected by real-time RT-PCR and western blotting. The ALP activity was analyzed in day 1, 3 and 7 post-transfection.

### Statistics

Quantitative results were expressed as mean±standard deviation (s.d.). All experiments were replicated three times. Independent samples *t*-test and one way analysis of variance (ANOVA) followed by Student–Newman–Keuls (S–N–K) post hoc analysis were performed with SPSS, v.20.0. Values were considered statistically significant at *P*<0.05.
