# Correction: IRS-1 increases TAZ expression and promotes osteogenic differentiation in rat bone marrow mesenchymal stem cells

**DOI:** 10.1242/bio.059772

**Published:** 2023-01-12

**Authors:** Na Wang, Peng Xue, Ziyi Li, Yukun Li

There were errors published in *Biol. Open* (2018) **7**, bio036194 (doi:10.1242/bio.036194).

In Fig. 2, the SiTAZ images in panel A were erroneously a duplication of the SiIRS-1 images. In addition, the control (non-transfected) panels were repeated in Fig. 2A and Fig. 4A. The corrected Fig. 2 is shown below.

**Fig. 2 (corrected).**
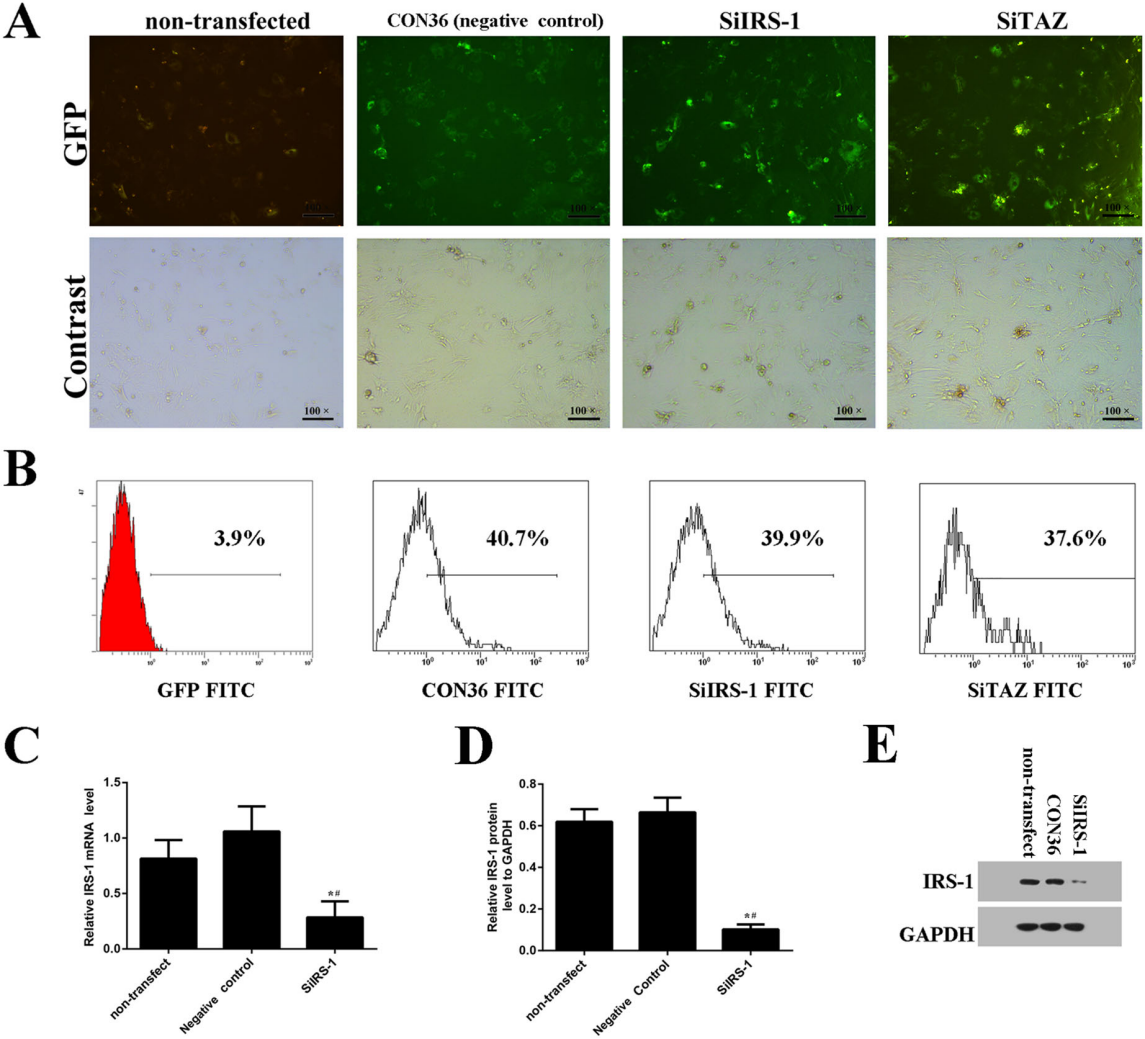
**The transfection efficiency of SiIRS-1, SiTAZ and the vacant plasmid as negative control during osteogenic differentiation of BMSCs.** (A) Seventy-two hours after transfection, several GFP+ cells were observed and counted under fluorescence microscopy. (B) Transfection efficiency was detected by the flow cytometer. (C-E) IRS-1 expression was analyzed using fluorogenic quantitative PCR (C,D) and western blotting (E). (s.d.±mean; n=3) *P<0.05 versus non-transfected group; ^#^*P*<0.05 versus negative control group.

Also, Fig 5J contained an incorrect image for the ‘IRS-1 over-expressed’ panel. The corrected Fig. 5 is shown below.

**Fig. 5 (corrected).**
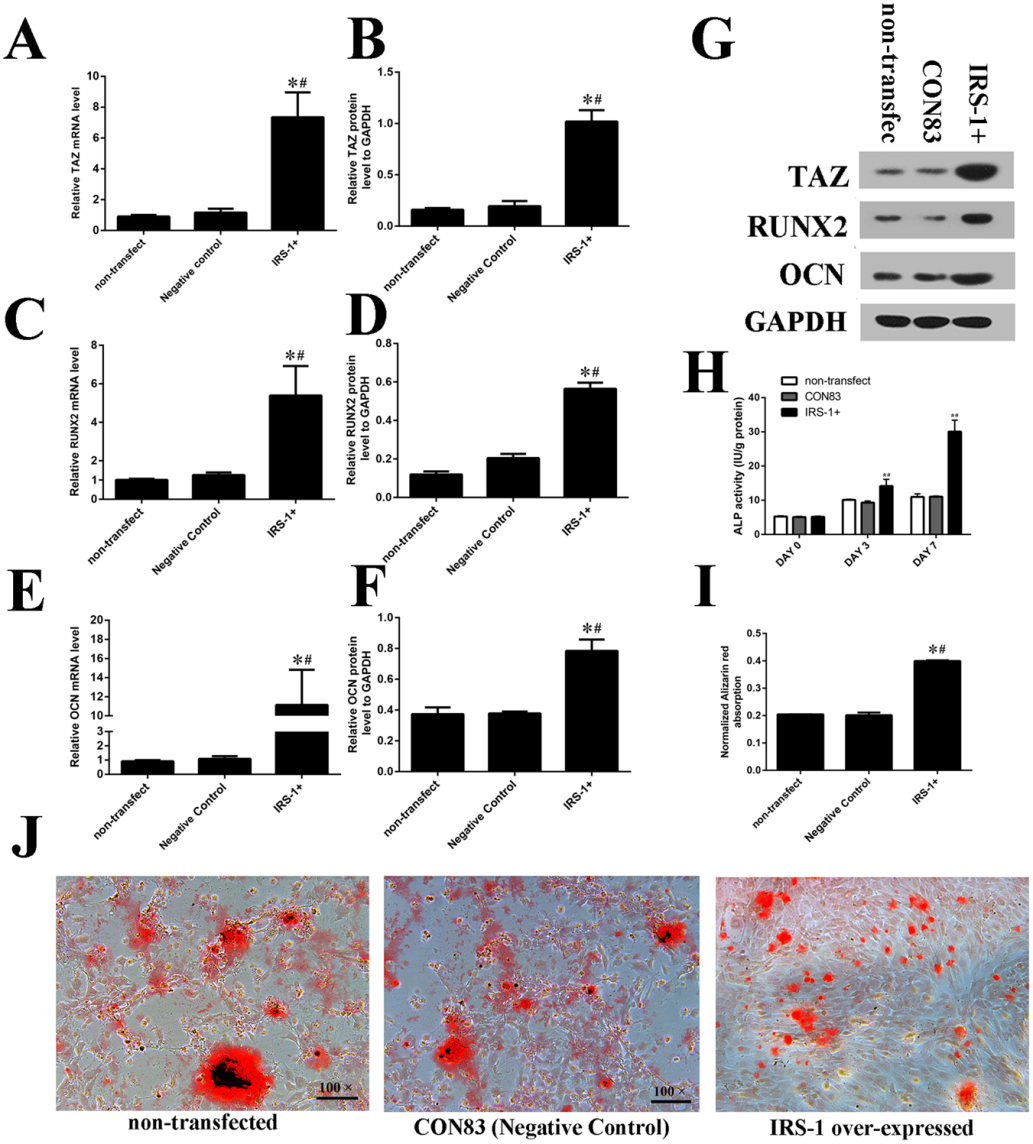
**IRS-1 overexpression promoted osteogenic differentiation as well as increasing TAZ expression.** (A-G) IRS-1 overexpression increased the TAZ, RUNX2 and OCN expression which were measured by fluorogenic quantitative PCR and western blotting. (H) The ALP activities were promoted in IRS-1+ group at 0, 3 and 7 days after osteogenic medium introduction. (I,J) Alizarin Red staining and the normalized Alizarin Red absorption were consistent with the ALP results which were presented after inducing osteogenic medium for 14 days. (s.d.±mean; n=3) *P<0.05 versus non-transfected group; ^#^*P*<0.05 versus negative control group.

The authors apologise to readers for these errors, which do not impact the results or conclusions of this paper.

